# Polymer Optical Fiber Goniometer: A New Portable, Low Cost and Reliable Sensor for Joint Analysis

**DOI:** 10.3390/s18124293

**Published:** 2018-12-06

**Authors:** Andressa Rezende, Camille Alves, Isabela Marques, Marco Aurélio Silva, Eduardo Naves

**Affiliations:** 1 Assistive Technology Lab., Faculty of Electrical Engineering, Federal University of Uberlandia, Uberlandia 38408-100, Brazil; camillealves@ufu.br (C.A.); isabela.marques@ufu.br (I.M.); eduardonaves@ufu.br (E.N.); 2 Faculty of Computer Science, Federal University of Uberlandia, Uberlandia 38408-100, Brazil; marcoams@algartech.com

**Keywords:** optical fiber sensors, wearable devices, joint angle measurement

## Abstract

The quantitative measurement of an articular motion is an important indicator of its functional state and for clinical and pathology diagnoses. Joint angle evaluation techniques can be applied to improve sports performance and provide feedback information for prostheses control. Polymer optical fiber (POF) sensors are presented as a novel method to evaluate joint angles, because they are compact, lightweight, flexible and immune to electromagnetic interference. This study aimed to characterize and implement a new portable and wearable system to measure angles based on a POF curvature sensor. This study also aimed to present the system performance in bench tests and in the measurement of the elbow joint in ten participants, comparing the results with a consolidated resistive goniometer. Results showed high repeatability of sensors between cycles and high similarity of behavior with the potentiometer, with the advantage of being more ergonomic. The proposed sensor presented errors comparable to the literature and showed some advantages over other goniometers, such as the inertial measurement unit (IMU) sensor and over other types of POF sensors. This demonstrates its applicability for joint angle evaluation.

## 1. Introduction

The quantitative measurement of articular motion is an important indicator of its functional state and for clinical and pathology diagnoses, and as an indicator of neurodegenerative disorders, which assists in the verification of joint and muscular limitation [1], and to determine the effectiveness of rehabilitation exercises [2]. Joint angle evaluation techniques can also be applied to improve sports performance [3] and provide feedback information for prostheses control [4]. The elbow joint motion has an important role in daily activities, and its amplitude can determine limitations, impairments, and can aid in medical diagnostics. The elbow articular motion can be analyzed during flexion–extension movement [5]. This measurement can be assessed through a variety of methods, such as goniometers, video-based techniques, inertial measurement units, and encoders, among others [4]. The most common is by using a goniometer, which can be made up of different types of sensors.

Currently, the main techniques employed in goniometers for this purpose are mechanical or electromechanical [6], which are normally implemented with a strain gauge and resistive potentiometers. The major disadvantages in using strain gauge sensors are their inaccuracy, while the potentiometer generated discomfort in some users, which can limit the natural movement of the member [7]. Another type of sensor used for this application is the inertial measurement unit (IMU), composed of gyroscopes, accelerometers and magnetometers. Although they are compact and lightweight, they present a high sensitivity with magnetic field interferences and can present high errors on the angle measurement [8,9]. A video tracking technique is also implemented, but is very expensive and time consuming [4].

Polymer optical fiber (POF) sensors are replacing conventional sensors due to several advantages, they are compact, lightweight, flexible, low cost, immune to electromagnetic interference [10], in addition to presenting high stability, resistance to impacts and high strain limits, which enable the fiber to bend in angles with great amplitude [11]. These advantages allow POF sensors to measure the magnitude of the range of movement of human joints, which has been applied in knee angle measurement [12] and for spinal posture monitoring [13]. Nowadays, several operating techniques have been proposed for POF sensors, among them, the intensity variation is the most common to measure joint angles [14].

The principle of intensity variation for angle estimation consists in the attenuation of the optical power in proportion to the curvature angle of the fiber [15], this attenuation can be measured with photodiodes. However, this attenuation is very low, so it is necessary to perform a lateral section, creating a sensitive zone, to improve the sensitivity of the fiber. The use of this technique allows the sensitivity control through the length and depth of the sensitive zone, as well as the roughness of its surface area [16]. The sensitive zone is on the convex side of the fiber, thus, with its curvature there is a reduction of reflections on the concave side, so that the rays escape, causing a signal attenuation, which increases with the curvature rise [17]. With different bending, it is possible to change the angle between the sensitive zone and the incident ray, changing the photodiode reading.

Some studies apply POF sensors to measure joint angles, such as Reference [18], in which the POF sensor was applied for gait analysis and compared with the video tracking method in four participants, to show the repeatability of the sensor. This study applied the sensor in the elbow joint for only one participant during the throwing movement of an object, which demonstrates the applicability of a POF sensor to measure the angle of this articulation. Gait was also analyzed in References [18,19], however, they did not compare this method with any other method. The studies presented had some limitations, such as not showing the repeatability of the sensor to measure the elbow joint angle in more than one participant and not performing a comparison with another method of joint measurement. Therefore, the aim of this study is to characterize and to implement a new portable, low-cost and wearable system to measure angles, based on a POF curvature sensor. In addition, we will present the system performance in tests with different angular velocities and its application for the measurement of elbow angles. We then compare the results with a consolidated resistive goniometer.

## 2. Experimental Analysis

A POF sensor is comprised of two modules: The emitter and the receiver of light. The emitter is a light emitting diode (LED) IF-E96 (Industrial Fiber Optics, Tempe, AZ, USA), that has a wavelength of 660 nm and is supplied by a 9-V battery. A resistance of 330 Ω was utilized in the circuit with the intent to limit the current in the LED. The receiver module is responsible for capturing the light from the fiber and it is composed of a photodiode IF-D91 (Industrial Fiber Optics, Tempe, AZ, USA) with a transimpedance amplifier with an adjustable gain. In all the experiments we used a multimode POF FB140-10 (Industrial Fiber Optics, Tempe, AZ, USA) with 160 mm, which is composed of three layers: Jacket, cladding and core. The jacket and cladding provide protection, while the core conducts the optical signals and is made of Polymethyl Methacrylate (PMMA), with 980 µm in diameter. An Arduino, at a sampling frequency of 10 Hz, made the acquisition of the signal response.

**Figure 1:**
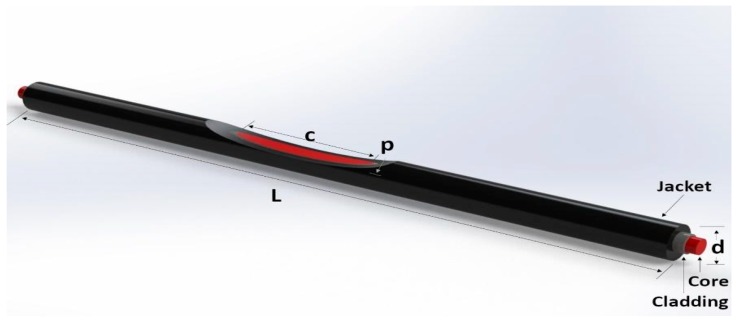
Polymer optical fiber (POF) curvature sensor with the sensitive zone. The optic fiber length is given by L and the optic fiber diameter is d. The sensitive zone length is represented by c and the depth of the cut in the fiber core is p.

The sensitive zone was created by polishing the material with sandpaper (400 grit size) connected to a drill, to ensure a smooth and continuous surface, since the sensitive zone parameters of section length, depth, and surface roughness provided different sensitivities and can influence sensor response. The value of the sensitive zone length and depth was fixed at 14 mm and 0.6 mm, respectively [16]. To obtain the desired section, the POF was positioned in a fixed support, which limits the drill to the desired length and depth specifications. The value of the sensitive zone depth was chosen because the fiber is very fragile in higher depths sections [15] and this is the lowest limit in a sensitive zone depth, since the POF has a polyethylene jacket and to reach the core the depth must be at least 0.6 mm. Once the sensitive zone is defined, the sensor maintains repeatability of the performance in the same velocity. Figure 1 presents the parameters of the POF and the sensitive zone.

In order to show the applicability and robustness of the POF’s sensor, two different setups were made: Test bench and joint angle measurement, respectively. In both tests the POF sensor was compared and correlated with a resistive goniometer, the most common and marketable electrogoniometer.

### 2.1. Test Bench

**Figure 2:**
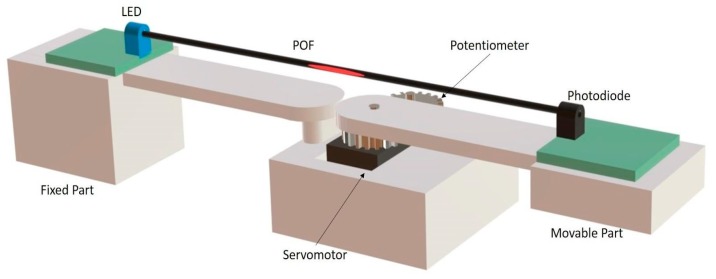
Test bench applied on the tests.

The experiment test bench was made by positioning the POF sensor in a prototype, as shown in Figure 2, which has a servomotor with position control, responsible for dynamic bending. The POF response was compared with the potentiometer response.

This test was performed in two ways: Quasi-static and dynamic. The tests were made with the aim of verifying the behavior and the performance of both sensors. The response curve of the POF sensor and the potentiometer were verified when they were bent. In the quasi-static test, the servomotor moved on sequential 10° steps in the 0–90° range and the results of the POF sensor and potentiometer were recorded. With these results a model to convert the sensors response (volts) in degrees was created. The dynamic test was made through bending the sensors in different angular velocities. The velocities used were 18, 36, 50, 80, 100, 140 and 200°/s and five repetitions were realized with 0–90° range, as this was applied to the conversion model obtained in the quasi-static test.

### 2.2. Joint Angle Measurement

To show the applicability of the POF sensor in joint angle measurement an experiment with 10 healthy participants (with ages between 19 and 31) was carried out. This study was approved by the National Committee of Ethics in Research, with a Certificate of Presentation for Ethical Appreciation, number 318,960.

**Figure 3:**
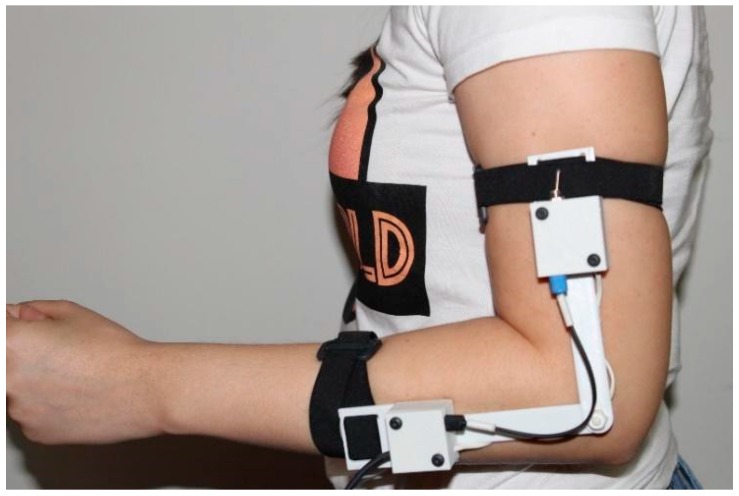
Positioning of the POF sensor at the elbow joint.

The sensor was embedded in a 3D printed structure and set up at the elbow joint of the participants, through elastic bands, in such a way that the sensitive zone was positioned exactly in the joint area, to give better ergonomics, as shown in Figure 3. For the experiment, the participants were instructed to realize the elbow flexion and extension movement five times, in an angular velocity at which they felt comfortable. The data, in degrees, from the potentiometer and the POF sensor were recorded at the same time during the movement.

## 3. Results and Discussion

### 3.1. Test Bench

**Figure 4:**
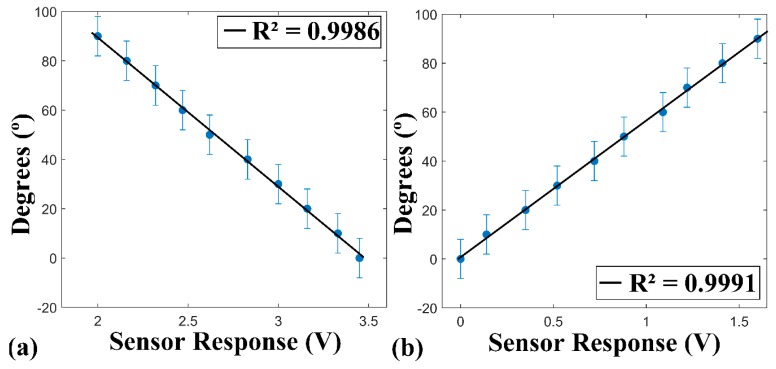
Results of quasi-static test for: (**a**) POF sensor and (**b**) potentiometer. The market point (blue points) are the values of the sensor response and the black line is the result of the linear regression.

The quasi-static test was realized with the intention of showing the POF sensor and the potentiometer’s behavior when they were submitted to an angle of 0–90°,with 10° steps between each measure. In Figure 4 the results of this test are presented.

It was observed that the POF sensor obtains a coefficient of determination (R^2^) of 0.9986, showing the linearity of the sensor in angles between the 0° and 90°. Similar results were found in Reference [20], which realized quasi-static tests in a test bench as well, with the same angles interval and which obtained a R^2^ of 0.9980 and in Reference [14], which achieved a R^2^ of 0.9917 in similar tests. The experiments made in References [15,21] showed a linearity of the POF sensor response in statics tests. This demonstrates that the purpose sensor obtains results compatible with the literature. The potentiometer utilized also presented a high coefficient of determination, 0.9991, following a linear standard, as expected.

**Figure 5:**
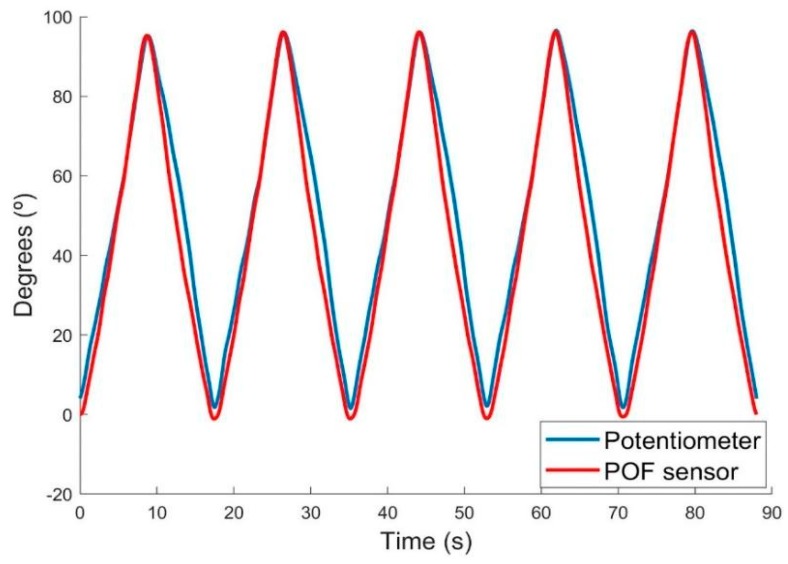
Results of the dynamic test for the 18°/s velocity. The blue line represents the potentiometer response and the red one represents the POF sensor response, in degrees.

The dynamic tests showed that the POF sensor has good repeatability between the cycles of extension and flexion and in the different velocities of bending, in other words, it exhibits a standard during all tests performed, with a mean deviation of 1.91° between cycles. Figure 5 presents the test results carried out in velocity 18°/s for the POF sensor and the potentiometer. The fiber behavior and the similarity in the response of both sensors can be observed.

With the objective of quantifying the similarity or the difference between these two variables, the difference was calculated between the POF sensor and the potentiometer response and the standard deviation of the difference for each velocity realized. The correlation among the measurements of the two sensors was also made. The results of these analyses are shown in Table 1.

The mean difference of all velocities was 6.33°and the mean standard deviation was 4.25. The highest difference was 7.93° for the highest velocity (200°/s) and the lowest was 4.95 for the velocity 50°/s. The highest standard deviation found was 4.54 for the velocity 36°/s and the lowest was 3.97 for the velocity 50°/s. In regard to the correlation coefficient, the results presented high positive correlation between the variables analyzed, providing 0.989 as the mean value. In a similar study, which realizes a dynamic analysis of a POF sensor and a potentiometer [15], similar correlations to those found in this experiment, of 0.993 to the velocity 85°/s, were shown.

The difference variations between velocities are not constant, due to the viscoelasticity of the POF material. The deformation of the viscoelastic material is related to stress and strain, which may vary over time, due to its molecular rearrangement [20]. Therefore, if the angular velocity changes constantly, there may be a cross sensitivity of the angle sensitivity to the angular velocity, which can lead to voltage variations, which result in different attenuation characteristics [12].

### 3.2. Joint Angle Measurement

The results obtained in the bench test (quasi-static and dynamic) demonstrated a high correlation coefficient, therefore, a test in participants was realized, with the intention of analyzing the sensor performance in a real scenario, verifying the behavior of the sensor in relation to anatomical particularities of the participants and comparing the sensor response with a potentiometer sensor when they were positioned at the arm of the participant.

**Figure 6:**
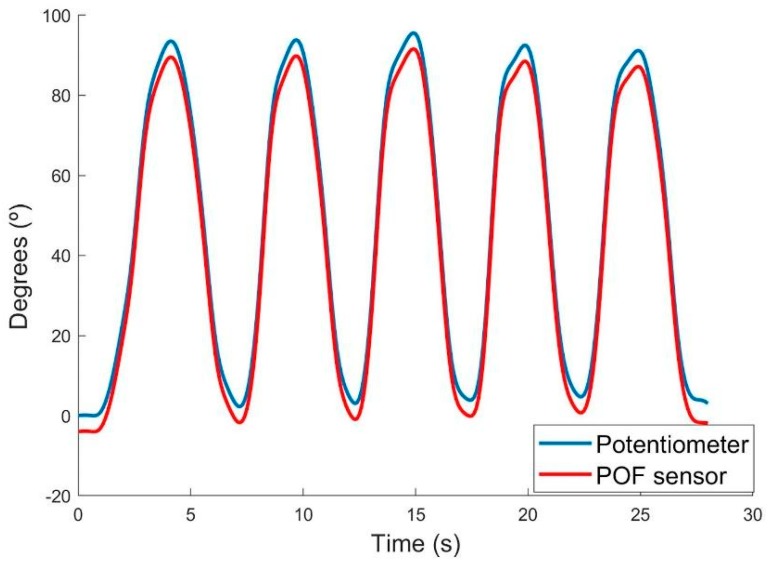
Results of the joint angle measurement for one participant. The blue line represents the potentiometer response and the red one represents the POF sensor response, in degrees.

To measure the elbow angle, five cycles were executed, where participants had to bend the arm in flexion and extension movements between the 0° and 90° in a desired velocity. Figure 6 presents the results of this test for one participant. It is possible to observe a great repeatability for both sensors between cycles, and similarity in their behavior.

In order to compare the results obtained by both sensors, and verify the similarity between angle measurements, the difference of the angle, the standard deviation and the correlation coefficient of these differences for each participant was calculated. Finally, the results were averaged for all participants, these values are shown in Table 2.

The mean difference for all participants was 5.31° and the standard deviation was 3.71°. A variation on the difference between participants was observed. This deviation is related to the human movement, since each participant performs the flexion and extension movements differently. In addition, as noted before, it is not possible to identify which sensor provided the highest accuracy during the experiment. The mean correlation coefficient was 0.987, indicating a strong positive relation between the two sensors.

The technique for measuring angles using the light intensity variation is a very feasible alternative, since it presents equivalent errors when compared to other sensors, like the Fiber Bragg grating type [5], with the advantage of being easy to produce compared to others based on optical fiber. In comparison with other commercial sensors, such as the IMU sensor [22], the proposed sensor presents lower errors, showing the possibility of its being used for the same purposes, furthermore, it does not present electromagnetic interference.

In the proposed sensor, a referral system is not used, i.e., the one used by Bilro et al. [14], composed of a fiber and a receptor, contributing to cost reduction and portability of the sensor. Furthermore, it is not necessary to perform a sensor calibration in each utilization. The sensor needs only one calibration in bench and after that can be utilized without another calibration, showing its wide applicability even outside of controlled environments.

Table 3 was developed in order to compare the main aspects of the proposed sensor with other sensors used to measure joint angles (commercial and from literature) and emphasize its advantages. The analyzed sensors were:

- Literature: IMU sensor developed by Vargas-Valencia et al. [22], the POF sensor developed by Arnaldo et al. [12] and the Fiber Bragg sensor developed by Umesh et al. [5].
- Commercial: The video tracking system of OptiTrack [23], the resistive goniometer of EMG System [24] and the strain gauge goniometer of Biometrics [25].

It should be emphasized that the errors found in this study can be reduced through compensation techniques, as done by Arnaldo et al. [4], which can improve the sensor response. These techniques will be implemented in future work. The reduction of errors would make the sensor more reliable for applications such as gait analysis. In future work it is also desired to increase the range of angulation to 140°, covering the entire angulation of the limbs. Nonetheless, the proposed sensor is presented as a low cost and portable option for wearable applications.

## 4. Conclusions

This paper presented the development and the application of a POF sensor for joint angle measurement. It was also possible to verify the performance of this sensor in comparison with the most used sensor for this purpose. The results showed that the sensors demonstrated a linear behavior, by the high correlation index in both the bench test (R^2^ = 0.989), and in the joint angle measurement test (R^2^ = 0.987). Another result considered to obtain more data on the performance of the POF sensor was the calculation of the error, which obtained less difference for the test applied in participants, confirming once more that the sensors have a similar behavior. Thus, for being portable, low-cost and having high reliability in joint analysis, the POF sensor is an interesting alternative as a new wearable system, as well as a resistive sensor that already has proven effectiveness, but with advantage over POF due to its better ergonomics.

## Figures and Tables

**Table 1:** Experimental results for dynamic tests realized in a test bench.

Angular Velocity	Difference (°)	Standard Deviation (°)	Correlation Coefficient
18°/s	5.75	4.18	0.989
36°/s	5.71	4.54	0.987
50°/s	4.95	3.97	0.989
80°/s	6.03	4.35	0.989
100°/s	6.48	4.48	0.989
140°/s	7.48	4.06	0.989
200°/s	7.93	4.17	0.989
Mean	6.33	4.25	0.989

**Table 2:** Experimental results for dynamic tests realized in the elbow for each participant.

	Difference (°)	Standard Deviation (°)	Correlation Coefficient
Participant 1	4.66	3.35	0.991
Participant 2	6.4	4.31	0.973
Participant 3	6.06	6.06	0.989
Participant 4	6.44	3.77	0.982
Participant 5	6.08	4.32	0.981
Participant 6	4.68	3.71	0.995
Participant 7	5.07	4.89	0.984
Participant 8	4.45	3.03	0.989
Participant 9	4.06	0.36	0.999
Participant 10	5.2	3.31	0.989
Mean	5.31	3.71	0.987

**Table 3:** Comparison between sensors for joint angle measurement.

	Low-Cost	Portable	No Calibration Required	Good Repeatability	No Electromagnetic Interferences	No Reference System
Vargas-Valencia et al. [22]	✓	✓		✓		✓
Arnaldo et al. [12]	✓	✓		✓	✓	
Umesh et al. [5]				✓	✓	
OptiTrack [23]			✓	✓	✓	
EMGSystem [24]		✓		✓	✓	✓
Biometrics [25]		✓	✓		✓	✓
Proposed sensor	✓	✓	✓	✓	✓	✓
